# Autophagy Protease ATG4D Facilitates Proliferation and Malignancy of Osteosarcoma Cells

**DOI:** 10.1096/fj.202501321RR

**Published:** 2025-08-29

**Authors:** Pei‐Feng Liu, Shan‐Wei Yang, Wen‐Hsin Yang, Shu‐Fang Huang, Ching‐Yang Tsai, Chien‐Jen Hsu, Ho‐Hsing Tseng, Cheng‐Hsin Lee, Chih‐Wen Shu

**Affiliations:** ^1^ Department of Biomedical Science and Environmental Biology Kaohsiung Medical University Kaohsiung Taiwan; ^2^ Department of Medical Research Kaohsiung Medical University Hospital Kaohsiung Taiwan; ^3^ Center for Cancer Research Kaohsiung Medical University Kaohsiung Taiwan; ^4^ Department of Orthopaedics Kaohsiung Veterans General Hospital Kaohsiung Taiwan; ^5^ Institute of BioPharmaceutical Sciences National Sun Yat‐Sen University Kaohsiung Taiwan; ^6^ Department of Medicine Chest Kaohsiung Armed Forces General Hospital Kaohsiung Taiwan; ^7^ Division of Gastroenterology, Department of Internal Medicine Yuan's General Hospital Kaohsiung Taiwan; ^8^ Innovation Center for Drug Development and Optimization National Sun Yat‐Sen University Kaohsiung Taiwan

**Keywords:** ATG4D, autophagy, osteosarcoma, prognosis

## Abstract

Osteosarcoma is one of the most prevalent malignant tumors in pediatric cancer, with survival rates remaining stagnant for over a decade, particularly among patients with metastatic disease. Thus, identifying novel therapeutic targets is critical for improving clinical outcomes. The *ATG4* family (*ATG4A*, *ATG4B*, *ATG4C*, and *ATG4D*) encodes proteases essential for autophagy, a process implicated in cancer progression and drug resistance. However, the role of ATG4 proteins in osteosarcoma remains unclear. This study showed that silencing *ATG4* family members using small interfering RNA (siRNA) induced G1‐phase cell cycle arrest and promoted cell death in osteosarcoma cells. Among them, *ATG4D* knockdown significantly impaired cell migration and invasion. Consistently, stable knockdown of *ATG4D* via short hairpin RNA (shRNA) reduced cell motility and tumorsphere formation. Moreover, *ATG4D* depletion enhanced autophagic markers, including LC3B‐II puncta and p62 protein levels, and sensitized osteosarcoma cells to starvation and chemotherapy‐induced cell death. In vivo, osteosarcoma cells harboring *ATG4D*‐targeting shRNA exhibited reduced tumor growth and elevated apoptosis in xenografted mice compared to control cells. Clinically, ATG4D protein expression was elevated in osteosarcoma tissues compared to normal bone cells, with higher levels correlating with poor overall survival, particularly in patients older than 10 years or with tumors located in the lower limbs. These findings suggest that ATG4D may serve as a potential diagnostic biomarker and therapeutic target for osteosarcoma.

## Graphical abstract

ATG4D appears to facilitate autophagy progression to degrade damaged organelles and ubiquitinated cargos in osteosarcoma cells, potentially through its involvement in autophagosome formation and the delipidation of LC3‐II. ATG4D‐mediated autophagy may contribute to cell cycle progression and enhance the migratory capacity of osteosarcoma cells. Clinically, elevated ATG4D expression correlates with poorer prognosis in patients with osteosarcoma, highlighting ATG4D as a potential therapeutic target for improving treatment outcomes.

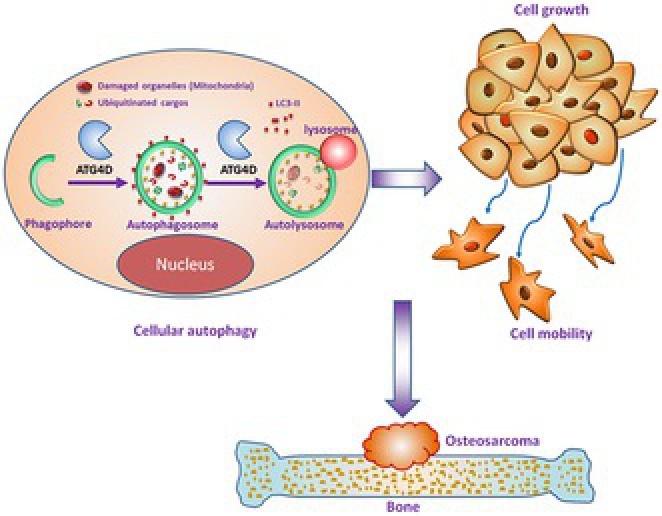

## 1 Introduction

Osteosarcoma is one of the most prevalent primary sarcomas affecting children and adolescents, accounting for approximately 20% of all bone cancer cases worldwide [1]. The global incidence of osteosarcoma is estimated to be 3–5 cases per million individuals annually [2]. A large cohort study involving over 4000 patients reports that the 1‐year, 5‐year, and 10‐year survival rates for individuals with appendicular osteosarcoma are 91.17%, 64.43%, and 58.58%, respectively [2]. Current standard treatment consists of surgical excision combined with neoadjuvant or adjuvant chemotherapy, which typically includes high‐dose methotrexate, doxorubicin, ifosfamide, and cisplatin [3]. Despite advancements in treatment strategies, the survival rate for osteosarcoma patients has remained unchanged over the past three decades [2, 4]. A large‐scale cohort study identified pulmonary metastases at diagnosis as a major adverse prognostic factor (hazard ratio [HR] = 2.34, 95% CI: 1.95–2.81) [2]. Disease relapse, primarily driven by metastasis and drug resistance, remains a significant challenge, particularly in patients with metastatic osteosarcoma, who exhibit a poor prognosis with a 5‐year survival rate below 30% [1]. These findings highlight an urgent need to identify novel therapeutic targets to improve treatment outcomes for osteosarcoma patients.

Autophagy is a cellular self‐degradation process that facilitates the removal of abnormal intracellular components. It plays a dual role in tumorigenesis and cancer progression [5]. While autophagy contributes to cellular homeostasis and genomic stability, thereby preventing tumor initiation, it is also activated in cancer cells under conditions such as nutrient deprivation, hypoxia [6], resistance to metastasis‐induced anoikis [7], and chemotherapy [8]. At least 38 autophagy‐related (*ATG*) genes have been identified as key regulators of autophagic signaling [9]. *Beclin‐1*, the mammalian homolog of *ATG6*, has been reported to undergo monoallelic deletion in human breast and ovarian cancers, supporting its role as a tumor suppressor [10]. Additionally, metformin, a drug commonly used to treat type II diabetes, has been shown to inhibit proliferation and induce cell death in various cancers, including osteosarcoma [11]. The association between metformin use and a reduced cancer incidence in diabetic patients further suggests a tumor‐suppressive role of autophagy in early‐stage cancer.

Conversely, numerous studies have demonstrated that elevated expression levels of ATG4B, ATG5, and Beclin‐1 in tumor tissues correlate with poor pathological outcomes in cancer patients [12, 13, 14]. Similarly, a positive correlation has been observed between the autophagy marker LC3B and metastasis in multiple cancer types, including breast cancer [15], melanoma [16], and glioblastoma [17]. In osteosarcoma, ULK1 expression is elevated in tumor tissues of patients with osteosarcoma compared to adjacent normal tissues [18]. Overexpression of ULK1 promotes cell proliferation and mobility of osteosarcoma cells [18]. Moreover, the proportion of LC3B‐positive samples increases progressively from pretreatment (34%) to posttreatment (50%) and metastatic cases (67%) in a cohort of 260 patients [19]. Chemotherapeutic agents, such as cisplatin (DDP), doxorubicin, and methotrexate, have been shown to upregulate Beclin‐1 expression in osteosarcoma cells, whereas Beclin‐1 knockdown suppresses osteosarcoma cell proliferation, metastasis, and invasion [20]. Notably, chemoresistant osteosarcoma cells often exhibit increased autophagy. Resistance to doxorubicin has been linked to the downregulation of miR‐143, which leads to autophagy activation and upregulation of ATG2B, Bcl‐2, and LC3‐II protein levels [21]. Furthermore, inhibition of autophagy through *Beclin‐1* and *LC3B* knockdown reduces both metastasis and chemoresistance in osteosarcoma cells [22]. These findings suggest that while autophagy functions as a tumor suppressor during early‐stage cancer development, it may promote tumor progression and therapy resistance in advanced cancer and under chemotherapeutic stress.

There are at least 38 *ATG* genes required for the core machinery of autophagy. It is important to investigate the association of the *ATG* genes with osteosarcoma to have a better understanding of the role of autophagy in osteosarcoma. The ATG4 family comprises a class of cysteine proteases essential for autophagy, consisting of four independent genes—*ATG4A* (*autophagin‐2*), *ATG4B* (*autophagin‐1*), *ATG4C* (*autophagin‐3*), and *ATG4D* (*autophagin‐4*)—in the human genome. These proteases are required for the cleavage and processing of mammalian ATG8 orthologues, including members of the LC3 and GABARAP/GABARAPL2 subfamilies, which are critical for autophagic activity [23, 24, 25]. The physiological roles of ATG4 in cancer have been studied using *Atg4* knockout mice. *Atg4b*‐deficient mice exhibit reduced autophagic flux, impairing protein secretion and leading to inner ear imbalance and decreased bone resorption [26]. Despite these defects, *Atg4b* knockout mice remain viable without severe abnormalities. However, *Atg4b* deficiency results in decreased skeletal muscle metabolism, resembling the phenotype observed in aged rodents [27], suggesting a potential role for autophagy in antiaging processes. In contrast, *Atg4c* knockout mice show minimal impact on autophagic flux but exhibit an increased risk of fibrosarcoma development [28]. Similarly, *Atg4d* knockout mice display cerebellar neurodegeneration and impaired motor coordination, highlighting its role in neuronal health [29].

In human cancers, hypomethylation of the *ATG4A* gene is associated with poor prognosis in ovarian cancer [30]. High expression of *ATG4A* is observed in ovarian tumor‐initiating cells, suggesting hypomethylation of *ATG4A* elevates *ATG4A* expression and promotes the stem properties and malignant phenotype of the cells. It has also been implicated in the survival of breast cancer stem cells [31]. Both *ATG4A* and *ATG4B* are upregulated in CD34^+^ chronic myeloid leukemia (CML) patients [32]. Overexpression of ATG4B has been linked to tumor progression and resistance to chemotherapy or radiation therapy [32, 33]. Notably, ATG4B is highly expressed in tumor tissues but shows minimal expression in adjacent normal tissues of colorectal and oral cancer patients [12, 33, 34, 35]. Silencing *ATG4B* significantly suppresses colorectal cancer (CRC) cell growth and enhances the sensitivity of HER2‐positive breast cancer cells to trastuzumab treatment [36]. In addition to ATG4B, ATG4C has been implicated in breast cancer tumorigenesis [37]. Furthermore, silencing *ATG4D* using miR‐101 or siRNA sensitizes breast cancer and HeLa cells to stress‐induced cell death [38]. However, *ATG4D* expression is lower in tumor‐adjacent normal tissues of CRC patients, suggesting a potential tumor‐suppressive role [39]. Despite these findings, the role of ATG4 family members in osteosarcoma remains largely unexplored, warranting further investigation.

## 2 Materials and Methods

### 2.1 Cell Culture and Transfection

Osteosarcoma U2OS and 143B cell lines were cultured in Dulbecco's Modified Eagle Medium (DMEM, Cat. no. 12100‐046, Thermo Fisher Scientific Inc.) supplemented with 10% fetal bovine serum (FBS, Cat. no. SH30071.03, Cytiva), 100 μg/mL streptomycin, 100 IU/mL penicillin, and 1% L‐glutamine at 37°C in a humidified atmosphere of 5% CO₂ and 95% air. For gene knockdown, cells were transfected with 5 nM small interfering RNA (siRNA) targeting *ATG4A* (Ambion, 35 623/121998), *ATG4B* (20 218/s23245/s23246), *ATG4C* (34 931/121984), and *ATG4D* (34 865/149022/s39787) for 72 h using Lipofectamine RNAiMAX (13778‐150, Thermo Fisher Scientific Inc.), following the manufacturer's protocol. To generate stable shRNA‐expressing cell lines, shRNA targeting *ATG4D* (TRCN0000073809) was obtained from The RNAi Consortium (TRC, Taiwan) and introduced into cells via lentiviral infection, followed by selection for stable integration. Additionally, pLVX‐LC3‐YFP plasmid (Addgene, 99 571) was used to assess autophagosome‐lysosome fusion via confocal microscopy, as described in the following methods.

### 2.2 Reverse Transcription‐Quantitative PCR

Total RNA was extracted from osteosarcoma cells using TRIzol reagent (Invitrogen, USA) following the manufacturer's protocol. Briefly, cells were resuspended in 1 mL of TRIzol reagent (Invitrogen; Thermo Fisher Scientific Inc.), and RNA was precipitated using 0.5 mL of isopropanol. The concentration, purity, and yield of total RNA were assessed using a Nanodrop 1000 spectrophotometer (Nanodrop Technologies Inc.). A total of 2 μg of RNA was further purified using an RNA extraction kit containing DNase I (Invitrogen, Thermo Fisher Scientific Inc.), then reverse‐transcribed into cDNA using oligo‐dT primers and SuperScript III Reverse Transcriptase (Invitrogen, Thermo Fisher Scientific Inc.) at 50°C for 50 min, followed by enzyme inactivation at 85°C for 5 min, according to the manufacturer's instructions. The synthesized cDNA was subsequently used for quantitative PCR (qPCR) analysis with gene‐specific primers, as detailed below. *ATG4A* forward 5′‐TGCTGGTTGGGGATGTATGC‐3′ and reverse 5′‐GCGTTGGTATTCTTTGGGTTGT‐3′, *ATG4B* forward 5′‐AGTTGGCGAAGGCAAGTCC‐3′ and reverse 5′‐CCACGTATCGAAGACAGCAAG‐3′, *ATG4C* forward 5′‐TAGAGGATCACGTAATTGCAGGA‐3′ and reverse 5′‐GTTGTCAAAGCTGAGCCTTCTAT‐3′, and *ATG4D* forward 5′‐GGAACAACGTCAAGTACGGTT‐3′ and reverse 5′‐CTCGCCCTCGAAACGGTAG using *ACTB* (*β‐actin*) (forward, 5‐′AGCGAGCATCCCCCAAAGTT‐3′ and reverse, 5′‐GGGCACGAAGGCTCATCATT‐3′) as normalization control. The gene expression was detected using a SYBR Green I assay and analyzed by real‐time PCR performed in StepOnePlusTM system (Applied Biosystems, Foster City, CA, USA).

### 2.3 Cell Viability Assay

Cells were transfected with siRNA for 72 h before assessing viability. CellTiter‐Glo (Cat. no. G7573, Promega Corporation) was added to the cells, and luminescence was quantified using a Fluoroskan Ascent FL reader (Thermo Fisher Scientific Inc.), with ATP levels serving as an indicator of cell viability. For the colony formation assay, cells were seeded in 12‐well plates at a density of 5 × 10^3^ cells/well and transfected with siRNA. Cells were then cultured in DMEM medium supplemented with 10% FBS at 37°C, with media refreshed every 3 days for 2 weeks. After incubation, cell colonies were fixed with 2% paraformaldehyde at room temperature for 15 min and subsequently stained with 0.25% crystal violet (Cat. no. C0775, Merck KGaA) in 20% ethanol at room temperature for 20 min. The stained cells were washed three times with PBS, and colonies with a diameter > 1 mm were counted and quantified using ImageJ software (https://imagej.net/ij/) in at least three independent experiments. The siRNA has been shown to achieve stable gene knockdown for at least 1 week [40], which allows us to observe the delayed growth effects in osteosarcoma cells. For the spheroid cell viability assay, cells were seeded at a density of 2.0 × 10^4^ cells/well in 24‐well NanoCulture plates (1.9 cm^2^, SCIVAX Corporation, Kanagawa, Japan) and cultured for 7 days until spheroid formation (diameter > 0.1 mm). Cell viability was assessed by adding CellTiter‐Glo 3D (Cat. no. G9681, Promega Corporation), and luminescence was quantified using a Fluoroskan Ascent FL reader (Thermo Fisher Scientific Inc.).

### 2.4 Flow Cytometry for Cell Cycle Analysis

The Knockdown cells were trypsinized, washed once with PBS, and fixed in 75% ethanol at −20°C overnight, following previously established protocols [41]. Fixed cells were then washed with PBS supplemented with 1% FBS and stained with 50 μg/mL propidium iodide (Sigma‐Aldrich) in the presence of 25 μg/mL RNase A (R6513, Sigma‐Aldrich) on ice for 30 min. Flow cytometric analysis and quantification were performed using the NovoCyte benchtop flow cytometer system (Agilent Technologies Inc., version 1.6.2; https://www.agilent.com/).

### 2.5 Cell Mobility Assay

For the invasion assay, 1.0 × 10^5^ cancer cells were seeded into the upper chamber of a Transwell insert pre‐coated with 50 μL of 0.5% Matrigel in DMEM supplemented with 1% FBS and incubated at 37°C for 30 min. The lower chamber was filled with 500 μL of DMEM containing 10% FBS to serve as a chemoattractant. Cells were allowed to invade through the Matrigel‐coated membrane for 24 h, after which they were fixed with 2% paraformaldehyde at room temperature for 15 min and stained with 0.25% crystal violet at room temperature for 30 min. For the migration assay, a Culture‐Insert 2 Wells system (IBIDI, USA) was used in 24‐well plates. A total of 1 × 10^6^ cells were seeded in 70 μL of DMEM supplemented with 10% FBS and incubated at 37°C overnight. The culture insert was then removed, and cells were allowed to migrate for 24 h. After incubation, cells were fixed with 2% paraformaldehyde at room temperature for 15 min, and the open area was measured. Images were captured using a light inverted microscope at 10× magnification.

### 2.6 Autophagy Assay

For immunoblotting assays, cells were lysed in RIPA buffer containing 1% NP‐40, 50 mM Tris–HCl (pH 7.5), 150 mM NaCl, 0.25% sodium deoxycholate, 1% SDS, a protease inhibitor cocktail, and a phosphatase inhibitor. Protein concentrations were determined using the bicinchoninic acid (BCA) assay, and 20 μg of total protein per sample was separated by 10% to 12% SDS‐PAGE and subsequently transferred onto nitrocellulose membranes. The membranes were blocked with 5% BSA at room temperature for 3 h, followed by overnight incubation at 4°C with primary antibodies diluted 1:1000, including LC3B (Arigo Biolaboratories, ARG55799), p62/SQSTM1 (Arigo Biolaboratories, ARG55040), and GAPDH (D16H11, Cell Signaling Technology, 5174). After primary antibody incubation, membranes were washed and probed with HRP‐conjugated secondary antibodies (Santa Cruz Biotechnology Inc., sc‐2004 and sc‐2005) at room temperature for 1 h. Protein bands were visualized using an enhanced chemiluminescence (ECL) kit, and images were analyzed with the Multi‐Function Gel Imaging System (TOPBIO, MGIS‐21‐C2‐6M). Protein expression levels were quantified using ImageJ software (National Institutes of Health, USA). Additionally, cells stably expressing YFP‐LC3 were utilized for the visualization of autophagosomes under confocal microscopy. Nuclear staining was performed using DAPI (4′,6‐diamidino‐2‐phenylindole), a blue‐emitting fluorescent compound.

### 2.7 Xenograft Mouse Model

The osteosarcoma 143B cells are isolated from the bone of female patients with osteosarcoma. Thus, female mice exhibit more consistent tumor engraftment and are less likely to exhibit hormonally influenced variability that may confound interpretation. Age‐matched female nude mice (6 weeks old) were used in assays for tumor growth and metastasis in a xenograft model. All experiments were performed as approved by the Institutional Animal Care and Use Committee at National Sun Yat‐sen University (No: 11221). 143B cancer cells (5 × 10^6^ in 0.05 mL of PBS) expressing luciferase were mixed with Matrigel (PBS: Matrigel = 1:1) and injected into mice (*n* = 5/group) subcutaneously. The tumor size was measured once a week with Vernier calipers and quantified with the equation (larger diameter) × (smaller diameter) 2 × π/6. The mice were sacrificed at Day 21 after transplantation with osteosarcoma cells to measure tumor weight.

### 2.8 Immunohistochemistry and Scoring

Tissue microarray (TMA) analysis of osteosarcoma was performed using commercially available TMAs obtained from SuperBiochips (CV2 Human, osteosarcoma) and US Biomax (BO481 and BO244d). Immunohistochemistry (IHC) staining was conducted to assess ATG4D expression on TMA or tumor tissues of xenografted mice. Tissue sections were subjected to antigen retrieval by immersing the blocks in 10 mM sodium citrate buffer (pH 6.0) and boiling at 125°C in a pressure boiler for 10 min. Endogenous peroxidase activity was blocked using 3% hydrogen peroxide at room temperature for 30 min. Tissue sections were incubated with anti‐ATG4D antibody (ab137621, Abcam) or active caspase‐3 (Asp175) (Cell signaling, 9661, 1:100), diluted in 2% BSA, and incubated overnight in a humidified chamber. Following TBS‐T washes, secondary antibody staining was performed using the Epredia UltraVision Quanto Detection System (TA‐125‐QHDX, Thermo Fisher Scientific Inc.), following the manufacturer's protocol. The slides were then rinsed with water and counterstained with hematoxylin (Sigma‐Aldrich, Massachusetts, USA) for 5 s. After air‐drying, the slides were mounted with coverslips and examined under a light microscope at 20× magnification.

The expression level of ATG4D in tissue samples was independently evaluated at least twice by orthopedic specialists Dr. Shan‐Wei Yang and Dr. Chien‐Jen Hsu. Staining intensity was assessed using a standardized three‐tiered scale: 1 (weak), 2 (moderate), and 3 (strong), as illustrated in Figure 6. The final expression score was determined according to the Allred scoring system, which combines a proportion score and an intensity score to yield a total ranging from 2 to 8. The ATG4D expression score of ≥ 7 was classified as “high” expression. For histological analysis, tissue sections were stained with hematoxylin and eosin (H&E) following standard procedures. Slides were deparaffinized, rehydrated, stained with hematoxylin for 5 min, briefly treated with 1% acid ethanol, counterstained with eosin for 1 min, dehydrated, and mounted using mounting medium.

## 3 Result

### 3.1 Silencing ATG4 Family Members Suppresses Cell Viability and Motility of Osteosarcoma Cells

**FIGURE 1:**
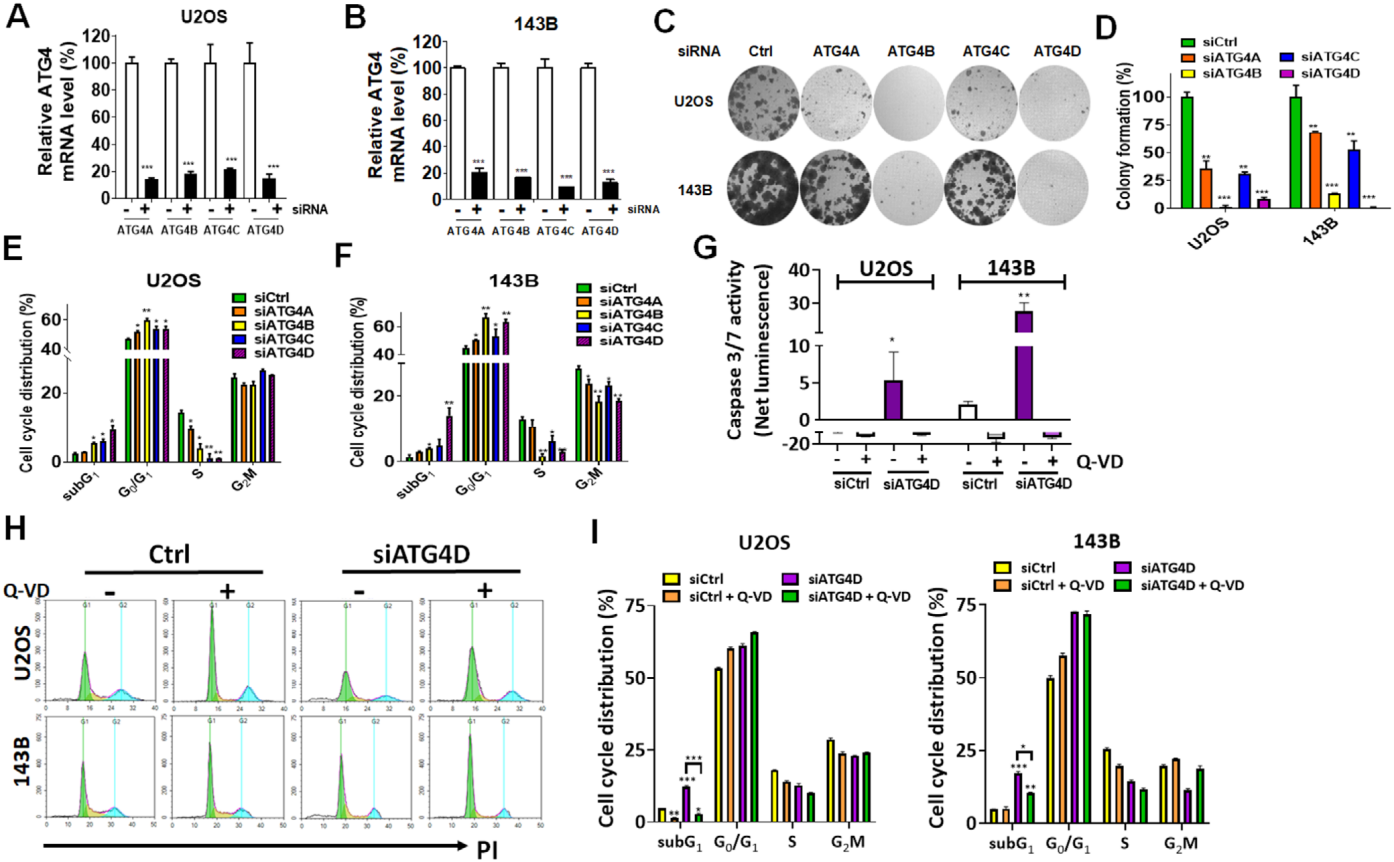
The effects of silencing ATG4 on proliferation and cell cycle distribution in osteosarcoma cells. (A) U2OS and (B) 143B osteosarcoma cells were transfected with 5 nM siRNA targeting *ATG4A, ATG4B, ATG4C*, or *ATG4D* for 48 h. The mRNA expression levels of each *ATG4* isoform were quantified using qPCR to confirm knockdown efficiency. (C) For the colony formation assay, cells were transfected with siRNA targeting *ATG4* family members and cultured with medium refreshed every 3 days until visible colonies formed. Representative colony images were acquired. (D) The number of colonies formed under each condition was quantified and statistically analyzed. (E) U2OS and (F) 143B cells were transfected with either scramble siRNA or siRNAs targeting individual *ATG4* isoforms as described above. After 72 h, cells were fixed and stained with propidium iodide, followed by flow cytometry to assess cell cycle distribution. (G) U2OS and 143B cells were transfected with scramble siRNA (si*Ctrl*) or siRNA against *ATG4D* (si*ATG4D*) for 72 h, with or without the pan‐caspase inhibitor Q‐VD (40 μM). Cells were lysed, and caspase‐3/7 activity was measured using the Caspase‐Glo 3/7 luminescent assay. (H) The same treated cells as (G) were analyzed for cell cycle distribution using PI staining and flow cytometry. (I) The effect of *ATG4D* knockdown, in the presence or absence of Q‐VD, on the subG1 cell population in U2OS and 143B cells was quantified. Statistical significance is indicated as follows: *p < 0.05, **p < 0.01, ***p < 0.001 compared to control cells transfected with scramble siRNA (si*Ctrl*).

**FIGURE 2:**
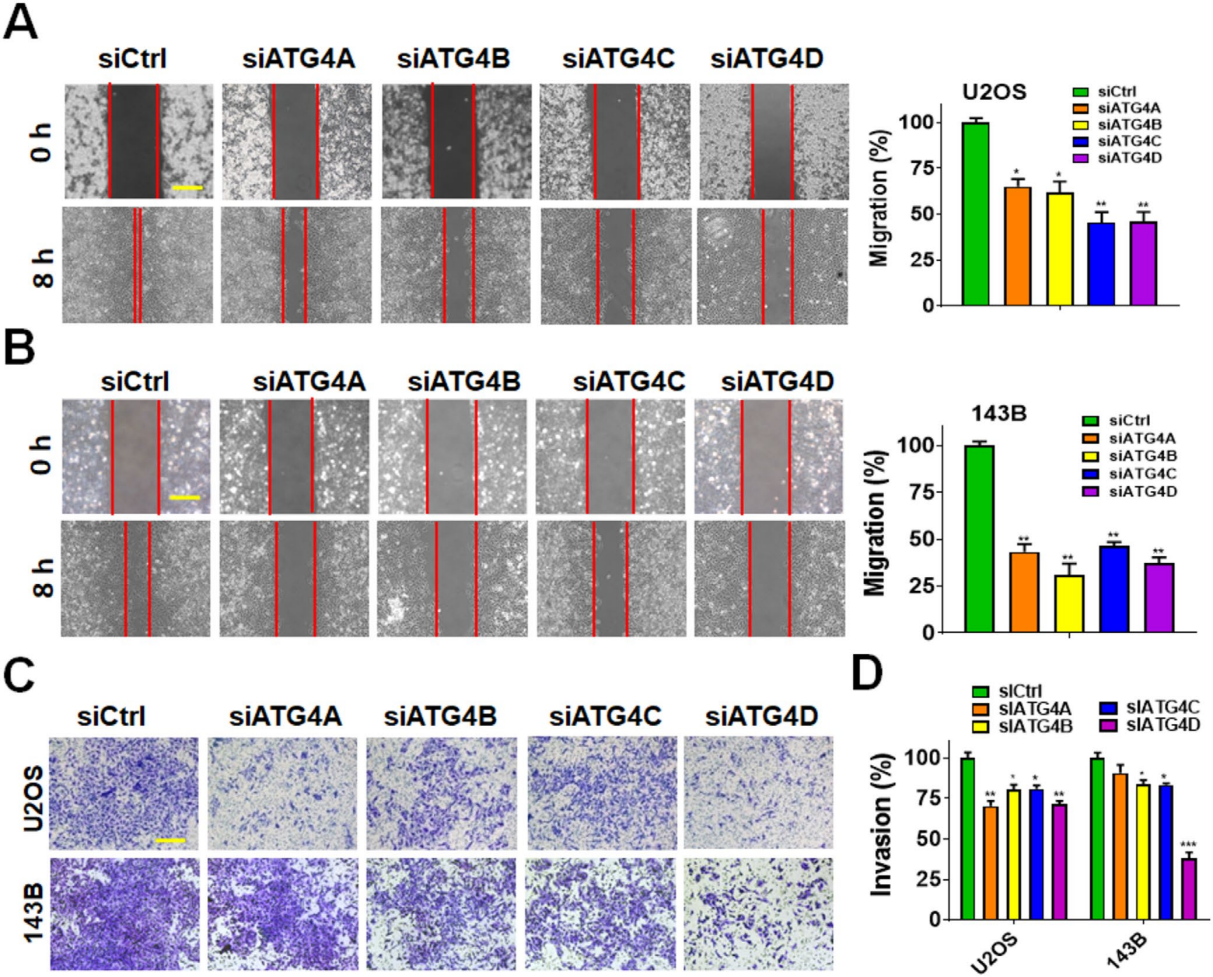
The effects of siRNA against ATG4s on cell migration and invasion in osteosarcoma cells. Human osteosarcoma cell lines (A) U2OS and (B) 143B were transfected with 5 nM scrambled siRNA (si*Ctrl*) or siRNAs targeting *ATG4A*, *ATG4B, ATG4C*, or *ATG4D*. Following transfection, equal amounts of cells were seeded into wound healing assay inserts to assess cell migration. Representative images were captured (left panel), and the wound closure area was measured using ImageJ software (right panel). Quantitative analysis was performed using GraphPad Prism, with si*Ctrl*‐transfected cells used as a reference control. Scale bar: 100 μm. (C) For the invasion assay, transfected cells were seeded in Matrigel‐coated transwell chambers. After incubation, cells that invaded to the lower surface of the membrane were fixed and stained with crystal violet. Representative images were acquired. Scale bar: 100 μm. (D) The number of invading cells was quantified using ImageJ, and invasion ability was compared to si*Ctrl*‐transfected cells. Statistical significance was denoted as: *p < 0.05, **p < 0.01, ***p < 0.001 compared to control cells transfected with scrambled siRNA (si*Ctrl*).

To investigate the role of ATG4 family members in osteosarcoma progression, U2OS and 143B osteosarcoma cell lines were transfected with siRNA targeting *ATG4A, ATG4B, ATG4C*, and *ATG4D*. The knockdown efficiency of each *ATG4* member was validated using real‐time PCR (Figure 1A,B), demonstrating at least an 80% reduction in mRNA expression levels. The impact of *ATG4* silencing on osteosarcoma cell proliferation was evaluated via colony formation assays (Figure 1C). Knockdown of any *ATG4* family member significantly inhibited colony formation in both U2OS and 143B cells, with the most pronounced effects observed in *ATG4B*‐ and *ATG4D*‐silenced cells (Figure 1D). To assess the effects of *ATG4* silencing on cell cycle progression and apoptosis, flow cytometric analysis was performed (Figure 1E,F). Knockdown of *ATG4A, ATG4B, ATG4C*, or *ATG4D* resulted in G1 phase cell cycle arrest. Additionally, *ATG4B*, *ATG4C*, and *ATG4D* silencing significantly increased the sub‐G1 population, indicative of cell death, in U2OS cells, while only *ATG4D* knockdown led to a notable increase in sub‐G1 cells in 143B. To determine whether apoptosis was involved in ATG4D‐mediated cell death, the specificity of si*ATG4D* was validated (Figure S1) and caspase‐3/7 activity was assessed (Figure 1G). Knockdown of *ATG4D* significantly reduced its mRNA expression levels in osteosarcoma cells (Figure S1). In contrast, the expression levels of *ATG4A, ATG4B*, and *ATG4C* remained unchanged. Silencing *ATG4D* led to a significant increase in caspase‐3/7 activity, which was attenuated by a pan‐caspase inhibitor (Figure 1G–I), indicating that *ATG4D* depletion induces caspase‐3/7‐mediated death in osteosarcoma cells. Additionally, *ATG4* knockdown significantly reduced the migratory capacity of U2OS and 143B cells (Figure 2A,B). Similarly, invasion assays demonstrated that silencing *ATG4A, ATG4B, ATG4C*, or *ATG4D* inhibited the invasive potential of U2OS cells (Figure 2C,D). In 143B cells, knockdown of *ATG4B, ATG4C*, and *ATG4D* significantly reduced invasion, whereas *ATG4A* silencing had no significant effect.

**FIGURE 3:**
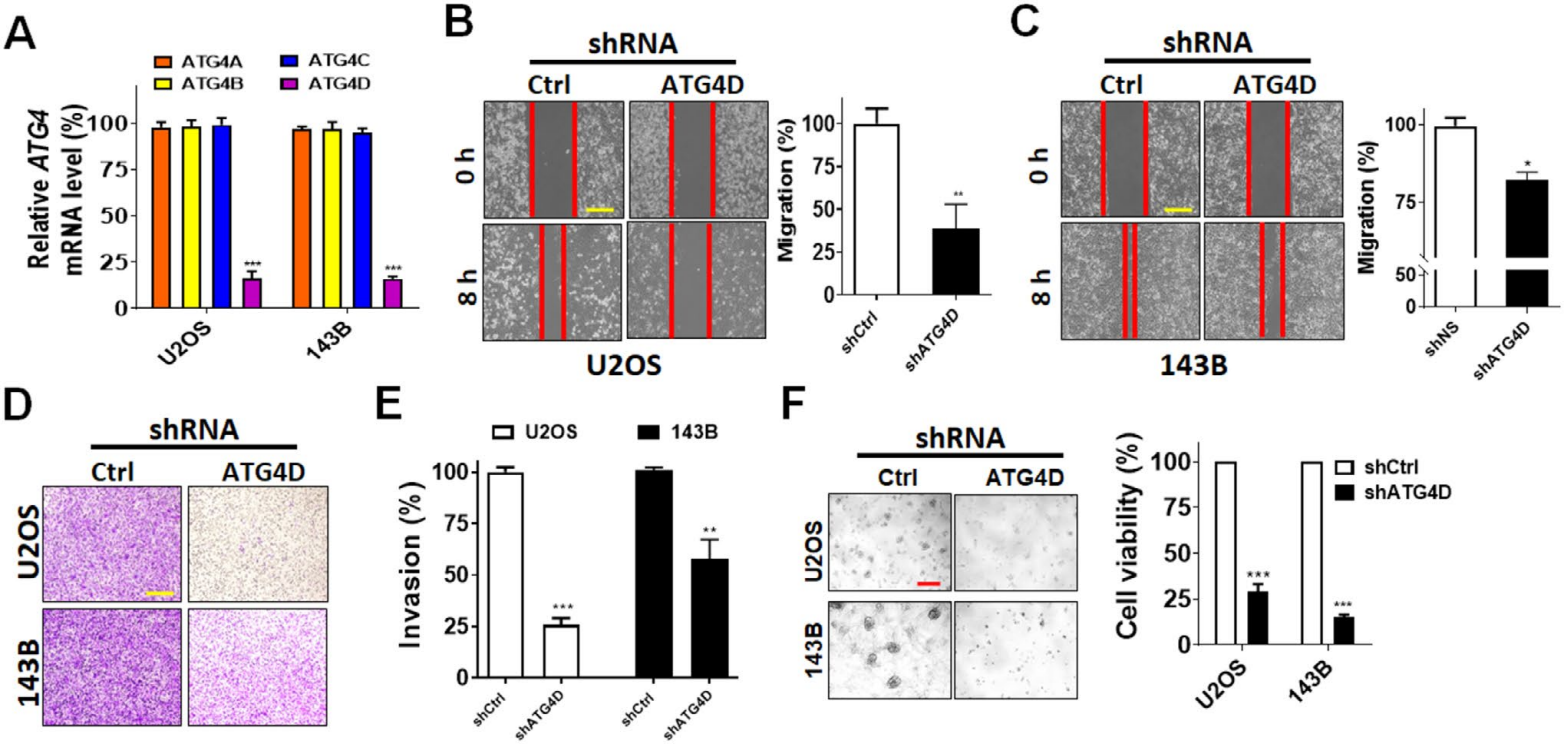
Effects of stable ATG4D knockdown on migration, invasion, and tumorsphere formation in osteosarcoma cells. (A) Human osteosarcoma U2OS and 143B cell lines stably expressing scrambled shRNA (sh*Ctrl*) or shRNA targeting *ATG4D* were harvested. The mRNA expression levels of each *ATG4* isoform were quantified using qPCR to confirm the knockdown specificity of si*ATG4D*. The migration capacity of *ATG4D*‐silenced U2OS (B) and 143B (C) cells was evaluated using a wound healing (gap closure) assay. Representative images were captured. Scale bar: 100 μm. (D) The invasive potential of stable *ATG4D* knockdown cells was assessed using Matrigel‐coated transwell invasion assays in both U2OS and 143B cells. Representative images of invaded cells on the lower surface of the membrane are shown. Scale bar: 100 μm. (E) Quantification of invaded cells was performed using ImageJ, with data expressed relative to sh*Ctrl*‐expressing cells. (F) The tumorsphere‐forming ability of *ATG4D*‐depleted U2OS and 143B cells was assessed using NanoCulture Plates (NCPs). Spheres were visualized under an inverted light microscope (magnification: 10×) and quantified using the 3D CellTiter‐Glo assay. Scale bar: 100 μm.

To further validate the inhibitory effects of silencing *ATG4D*, stable knockdown was achieved using shRNA targeting *ATG4D* (Figure 3). The knockdown specificity and efficiency were confirmed via real‐time PCR (Figure 3A). Stable *ATG4D* knockdown significantly impaired migration (Figure 3B,C) and invasion (Figure 3D,E) in both U2OS and 143B cells, with the most pronounced effects observed in U2OS cells. In addition, compared to two‐dimensional culture, three‐dimensional tumor cell culture can better mimic the complex in vivo microenvironment, including conditions like nutrient and oxygen deprivation [42]. Moreover, autophagy plays a crucial role in enabling cancer cell survival under conditions of starvation and hypoxia [43]; thus, to evaluate if silencing *ATG4D* genes may disrupt this survival mechanism and promote cancer cell death, osteosarcoma cells transduced with nontargeting shRNA or *ATG4D*‐targeting shRNA were cultured in a 3D NanoCulture Plate (NCP). The results demonstrated that *ATG4D* knockdown suppressed sphere formation and cell viability in both U2OS and 143B cells, with the most substantial effect observed in 143B cells (Figure 3F).

### 3.2 Silencing *ATG4D* Inhibits Autophagy and Chemoresistance in Osteosarcoma Cells

**FIGURE 4:**
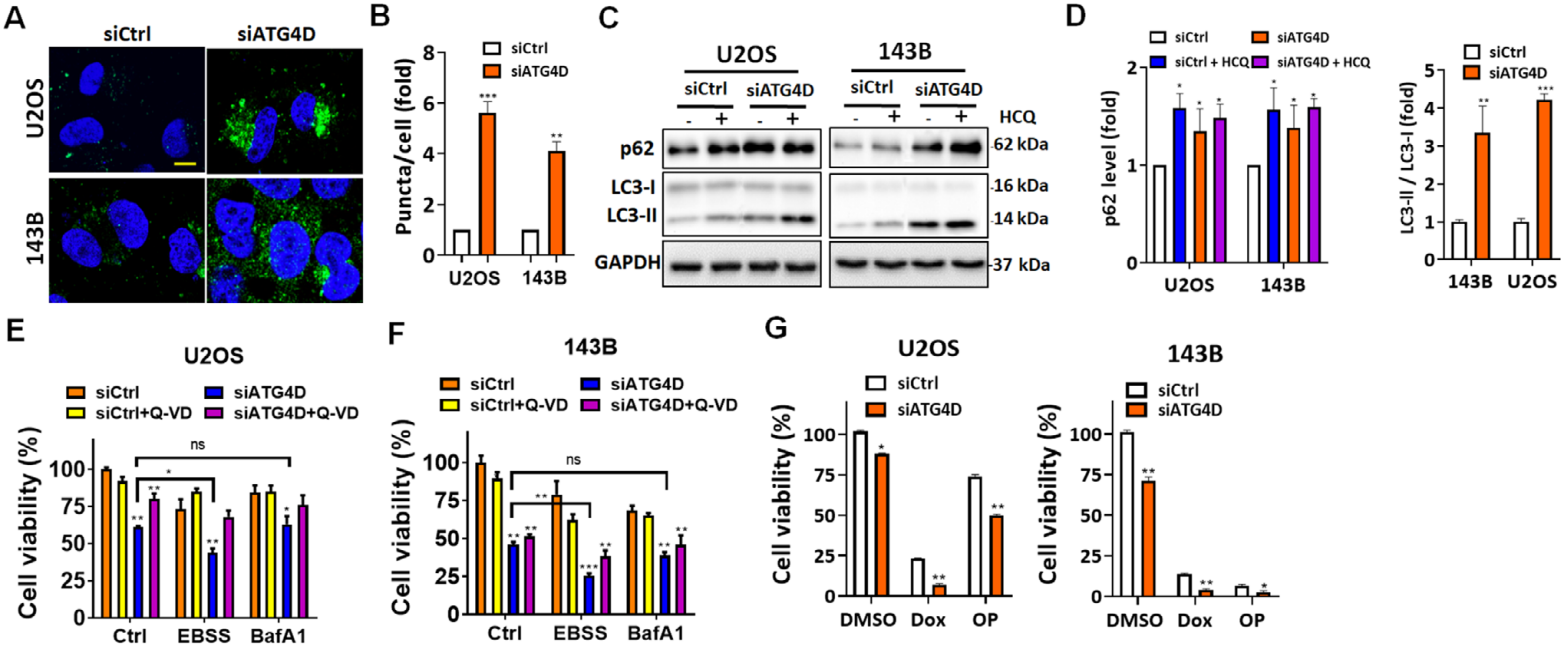
The effects of ATG4D‐mediated autophagy on cell viability under stress conditions in osteosarcoma cells. (A) U2OS and 143B osteosarcoma cells stably expressing YFP‐LC3 were transfected with 5 nM scrambled siRNA (si*Ctrl*) or siRNA targeting *ATG4D* for 72 h. Cells were fixed and counterstained with DAPI to visualize YFP‐LC3 puncta, indicative of autophagosome formation. Scale bar: 10 μm. (B) Quantification of autophagosome puncta per cell was performed, with data expressed as fold change relative to si*Ctrl*‐transfected cells. (C) U2OS and 143B cells transfected with si*ATG4D* were treated with the autophagy inhibitor hydroxychloroquine (HCQ) for 3 h. The levels of LC3‐I, LC3‐II, and p62 were examined by immunoblotting to assess autophagic flux. (D) Quantification of the LC3‐II/LC3‐I ratio and p62 accumulation was performed, and results were expressed as fold change relative to control cells. (E) U2OS and (F) 143B cells were cultured under either autophagy‐inducing conditions (EBSS) or autophagy‐inhibiting conditions (100 nM Bafilomycin A1) for 24 h. Cell viability was determined using the CellTiter‐Glo assay. (G) U2OS and 143B cells were transfected with 5 nM si*Ctrl* or si*ATG4D* for 72 h and subsequently treated with doxorubicin (1 μM) or oxaliplatin (50 μM) for 24 h. Cell viability was measured using the CellTiter‐Glo assay. *Data are presented as mean ± SD. Statistical significance: *p < 0.05, **p < 0.01, ***p < 0.001 compared with si*Ctrl*‐treated cells. BafA1, Bafilomycin A1; EBSS, Earle's balanced salt solution.

Although ATG4 family members are important for autophagosome formation and maturation [44, 45], the specific role of ATG4D in autophagy regulation in cancer cells remains unclear. This uncertainty may be attributed to its relatively low protease activity compared to other family members, such as ATG4A and ATG4B [24, 25]. To investigate the impact of ATG4D depletion on autophagy, osteosarcoma cells stably expressing YFP‐LC3 were generated and examined for autophagosome formation. *ATG4D* knockdown led to a significant increase in LC3‐positive puncta in both U2OS and 143B cells (Figure 4A,B). Similarly, LC3 and the autophagy adaptor protein p62 were upregulated in *ATG4D*‐silenced osteosarcoma cells, resembling the effects observed upon hydroxychloroquine (HCQ) treatment, an autophagy inhibitor (Figure 4C,D). These findings suggest that ATG4D deficiency impairs autophagic flux, supporting its role in removal of ATG8 from autophagosomes or ATG8ylation [29, 46]. Furthermore, autophagy serves as a cytoprotective mechanism under nutrient deprivation [47]. The decrease in cell viability following *ATG4D* silencing was further exacerbated under starvation conditions (EBSS treatment, Figure 4E,F). However, treatment with an autophagy inhibitor did not further reduce cell viability, indicating that ATG4D depletion alone may sufficiently impair autophagy‐dependent survival mechanisms. Additionally, *ATG4D* knockdown enhanced the cytotoxic effects of chemotherapeutic agents, including doxorubicin and oxaliplatin, in osteosarcoma cells (Figure 4G). These findings suggest that autophagy serves as a survival mechanism in stressed conditions, and ATG4D depletion may enhance stress‐induced cell death.

### 3.3 ATG4D Promotes Tumor Growth in Vivo and Is Associated With Poor Prognosis

**FIGURE 5:**
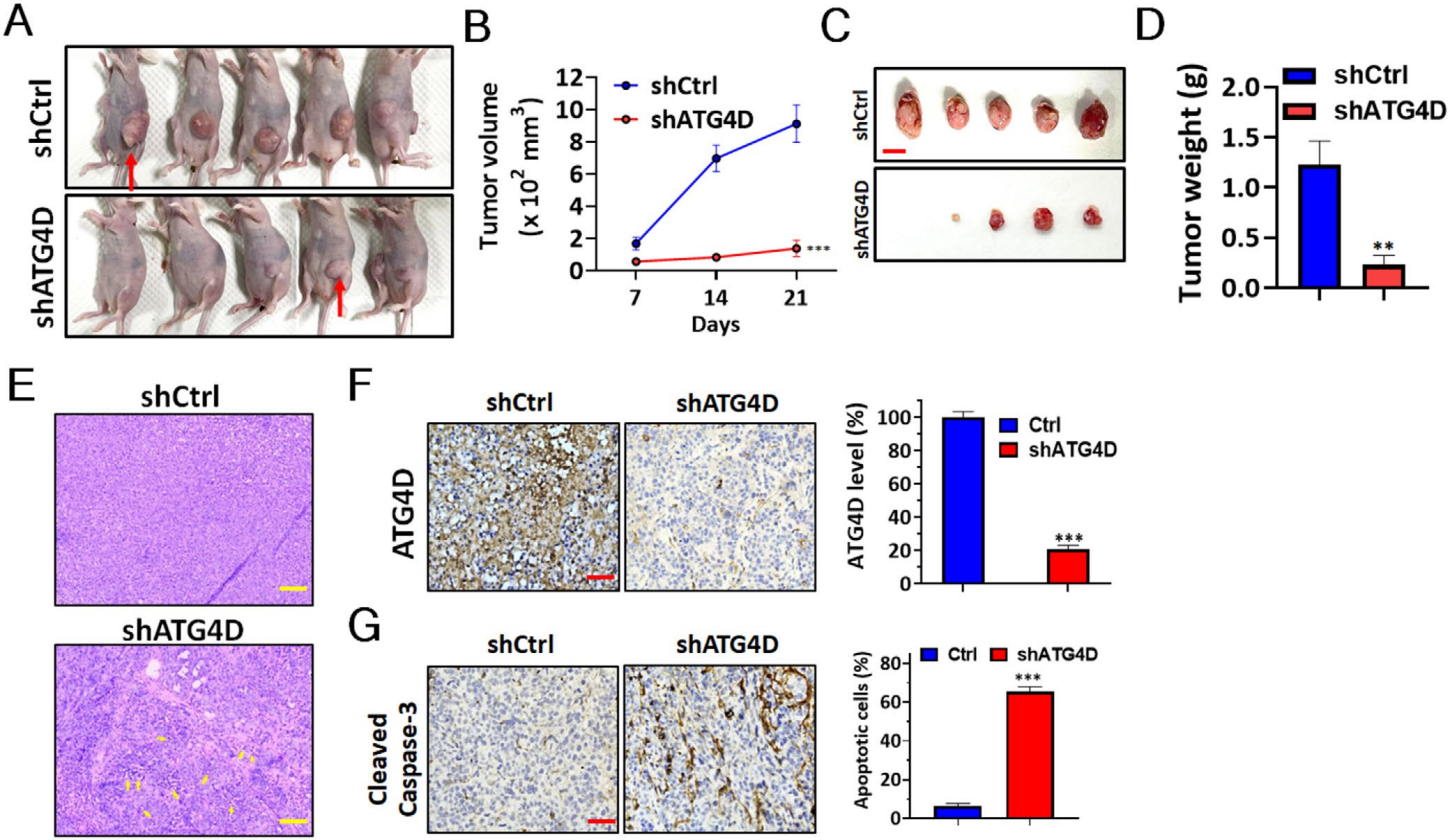
The effects of ATG4D knockdown on tumor growth in vivo. (A) Human osteosarcoma 143B cells stably expressing either nontargeting shRNA (sh*Ctrl*) or shRNA targeting *ATG4D* (sh*ATG4D*) were subcutaneously xenografted into nude mice (*n* = 5 per group). Tumor formation was monitored for 21 days, and representative images of tumor‐bearing mice are shown. Red arrows indicate representative xenografted tumors. (B) Tumor volumes were measured weekly and plotted over time. (C) At the experimental endpoint, tumors were excised from euthanized mice and imaged. Scale bar: 1 cm. (D) Excised tumors were weighed, and tumor weights were presented as mean ± SD. (E) Representative images of HE staining for tissue sections from 143B cells xenografted tumor that developed in mice. The dead cells are indicated (arrowhead) in tumors of sh*ATG4D*. Scale bar, 200 μm. (F) The ATG4D expression was determined with immunohistochemistry as shown in the left panel. The protein level of ATG4D was quantified by staining intensity in the right panel. (G) The cleaved caspase‐3‐positive cells were counted as apoptotic cells as shown in the left panel. The quantified results were obtained from at least 500 cells and are shown in the right panel. Scale bar: 50 μm. *Statistical significance: *p < 0.05, **p < 0.01, ***p < 0.001 compared with the sh*Ctrl* group.

To investigate the role of ATG4D in vivo, a tumor xenograft mouse model was established using osteosarcoma cells transduced with nontargeting shRNA or shRNA against *ATG4D* (Figure 5). Tumor volume in mice xenografted with osteosarcoma cells expressing *ATG4D*‐targeting shRNA exhibited a significant weekly decrease compared to tumors derived from nontarget osteosarcoma cells (Figure 5A,B). Consistently, the tumor weight in sh*ATG4D* osteosarcoma cells was significantly lower than that in the control (shCtrl) group (Figure 5C,D). Tumor tissues were further evaluated by H&E staining (Figure 5E) and IHC analysis (Figure 5F,G). Histological examination revealed that tumors derived from sh*Ctrl* osteosarcoma cells exhibited higher cell density and homogenous nuclear morphology, whereas those from sh*ATG4D* cells displayed increased cell death (Figure 5E). The sh*ATG4D* tumors exhibited a necrosis‐like island architecture. The cells displayed nuclear size variability, chromatin condensation, nuclear fragmentation, and hypereosinophilic cytoplasm. These morphological features are indicative of apoptosis in the tumor sections. Consistently, IHC staining demonstrated elevated levels of active caspase‐3 in sh*ATG4D* tumors compared to sh*Ctrl*, supporting the proapoptotic effects observed in vitro models (Figure 5F,G).

**FIGURE 6:**
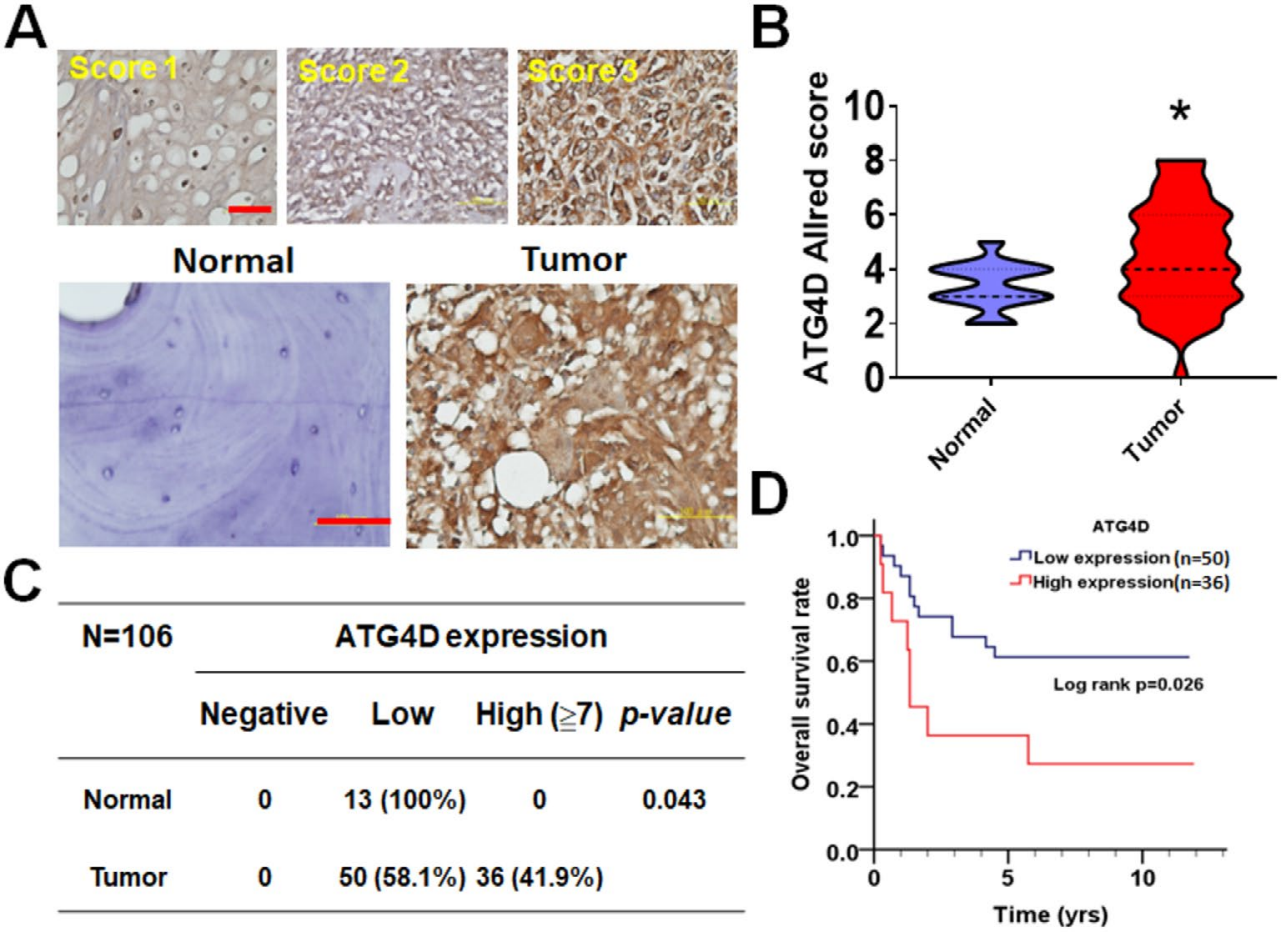
The protein expression level of ATG4D in tissues of osteosarcoma patients. (A) Tissue microarrays from osteosarcoma patients were subjected to immunohistochemical staining to assess ATG4D protein expression. Expression levels in normal osteoblasts/osteoclasts and malignant osteosarcoma tissues were evaluated based on the scoring criteria illustrated in the upper panel. Representative immunohistochemical images of ATG4D staining in normal and malignant tissues are shown in the lower panel. Scale bar: 100 μm. (B) ATG4D expression was quantified using the Allred scoring system, and results were analyzed using GraphPad Prism 5.0. (C) Based on Allred scores, ATG4D expression was categorized as negative (score = 0), low (score < 7), and high (score ≥ 7). Comparative expression levels between normal and osteosarcoma tissues are presented. (D) The correlation between ATG4D expression levels in tumor tissues and overall survival of osteosarcoma patients was analyzed using the Kaplan–Meier survival analysis.

**FIGURE 7:**
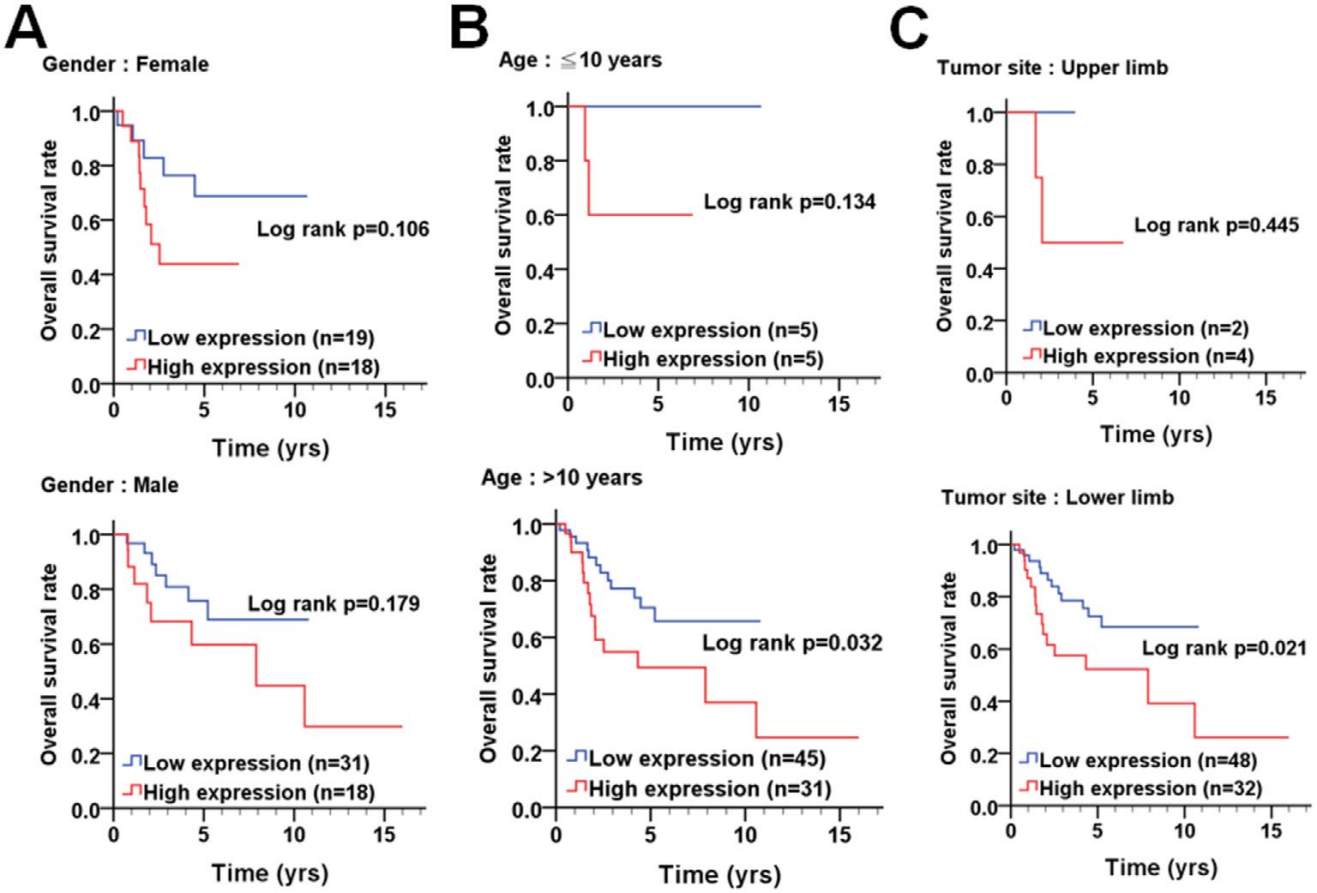
The correlation of ATG4D level with the survival curve of sarcoma patients according to clinicopathological outcomes. The association between ATG4D expression levels and overall survival was evaluated in sarcoma patients stratified by clinicopathological features using the Kaplan–Meier survival analysis. (A) Patients were grouped by sex (female [upper panel] and male [lower panel]) to assess the correlation between ATG4D expression and survival outcomes. (B) The impact of ATG4D expression on survival was analyzed in patients younger than 10 years and those aged 10 years or older. (C) Patients were also stratified based on tumor location (upper limb vs. lower limb) to investigate the relationship between ATG4D expression and overall survival.

To assess the clinical significance of ATG4D in osteosarcoma, its protein expression levels were analyzed using a TMA‐containing tumor samples from osteosarcoma patients (Figure 6A). Immunohistochemical (IHC) staining revealed that ATG4D protein levels were significantly elevated in tumor tissues compared to normal osteoblasts or osteoclasts (Figure 6B). The distribution of ATG4D protein expression in normal and tumor tissues was further analyzed, indicating that 36 tumor tissues exhibited high ATG4D levels, while 50 tumors displayed low ATG4D expression (Figure 6C). Kaplan–Meier survival analysis indicated that osteosarcoma patients with high ATG4D expression had significantly poorer overall survival (*p* = 0.026, Figure 6D). Further subgroup analysis stratified patients by gender, age, and tumor location to assess the correlation between ATG4D expression and clinical outcomes (Figure 7 and Table 1). While both male (*p* = 0.179) and female (*p* = 0.106) patients with high ATG4D expression showed a trend toward shorter overall survival, the results were not statistically significant (Figure 7A). However, patients older than 10 years with high ATG4D expression exhibited significantly worse overall survival (*p* = 0.032, Figure 7B; CHR: 2.27, 95%, Table 1), whereas no significant association was observed in patients younger than 10 years (*p* = 0.134). Additionally, elevated ATG4D levels in lower limb osteosarcoma tumors correlated with poor overall survival (*p* = 0.021, Figure 7C; CHR: 2.40, 95%, Table 1), whereas ATG4D expression in upper limb tumors showed no significant impact on prognosis. These findings suggest that ATG4D may serve as a prognostic marker in specific osteosarcoma subgroups. Although ATG4D expression was not significantly associated with poor survival in younger patients (< 10 years) or in upper limb tumors, this may be attributed to the limited sample size in each subgroup. Therefore, larger cohort studies are required to further elucidate the prognostic significance of ATG4D in osteosarcoma.

**TABLE 1:** Impact of ATG4D expression levels on overall survival by the different demographic and clinicopathologic factors with sarcoma.

Variable		No. (%)	CHR (95% CI)	*p value
ATG4D	Low	50 (58.1)	1.00	
	High	36 (41.9)	2.44 (1.16–5.14)	**0.019**
Sex				
Female	Low	19 (51.4)	1.00	
	High	18 (48.6)	2.43 (0.80–7.37)	0.117
Male	Low	31 (63.3)	1.00	
	High	18 (36.7)	2.01 (0.71–5.65)	0.187
Age, years				
≦ 10	Low	5 (50.0)	1.00	
	High	5 (50.0)	73.97 (0.00–6678390.40)	0.460
> 10	Low	45 (59.2)	1.00	
	High	31 (40.8)	2.27 (1.05–4.89)	**0.037**
Tumor site				
Upper limb	Low	2 (33.3)	1.00	
	High	4 (66.6)	30.08 (0.00–44789592.59)	0.460
Lower limb	Low	48 (60.0)	1.00	
	High	32 (40.0)	2.40 (1.11–5.16)	**0.025**

Abbreviations: CHR, crude hazard ratio; CI, confidence interval.

* *p* values were estimated by Cox's regression and bold values represented as statistical significance (< 0.05).

## 4 Discussion

Osteosarcoma is a highly aggressive malignancy that predominantly affects children and adolescents [48]. Despite significant advances in cancer research, there has been little improvement in long‐term survival rates for osteosarcoma over the past several decades, underscoring the urgent need to identify effective therapeutic targets for this disease. ATG4, a key protease involved in autophagy signaling, has been closely linked to tumor progression. The upregulation or downregulation of various ATG4 family members has been associated with different cancer types [12, 32, 33, 39, 49, 50]; however, the specific role of ATG4 in osteosarcoma remains unclear. In this study, we report the following findings: (i) Knockdown of *ATG4* family members resulted in cell cycle arrest at the G1 phase and an increase in the subG1 proportion, with the most pronounced effects observed in *ATG4D*‐silenced osteosarcoma cells. Pan‐caspase inhibition diminished the elevated caspase‐3/7 activity and reduced the subG1 population in these cells; (ii) Knockdown of *ATG4D* inhibited migration, invasion, and tumorsphere formation in osteosarcoma cells; (iii) Silencing *ATG4D* led to an increase in LC3 puncta, as well as the accumulation of LC3‐II and p62, suggesting a disruption in autophagic flux. *ATG4D*‐silenced osteosarcoma cells exhibited heightened sensitivity to starvation and chemotherapeutic drugs when compared to cells treated with scrambled siRNA; (iv) Silencing *ATG4D* inhibited tumor formation in xenografted mouse models. Additionally, ATG4D protein expression was found to be elevated in tumor tissues and correlated with poor prognosis in patients with osteosarcoma. Our findings suggest that ATG4D plays a critical role in regulating osteosarcoma progression and may serve as a potential therapeutic target for improving treatment outcomes in osteosarcoma patients.

The high expression of ATG4B has been correlated with shorter survival in patients with pancreatic ductal adenocarcinoma (PDAC) [51]. Knockout of *Atg4b* resulted in reduced cell proliferation, accompanied by decreased levels of GABARAP and GABARAPL1 in PDAC cells. Moreover, *Atg4b* and *Atg4a* double knockout delayed the G1‐S phase transition and mitosis during the cell cycle in PDAC cells. Silencing ATG4B also led to cell cycle arrest at the G1‐S phase transition in both CRC and glioblastoma cells [33, 52]. Additionally, G1 phase arrest was observed in glioblastoma cells expressing the ATG4B C74A mutant [52]. Furthermore, silencing ATG4B using siRNA enhanced the cytotoxic effects of irinotecan and oxaliplatin in CRC cells [12]. Knockdown of *ATG4A* with shRNA potentiated 4‐hydroxytamoxifen‐induced apoptosis in breast cancer cells [49]. In various cancer types, ATG4B inhibitors have shown significant synergy with chemotherapeutic drugs, including Azalomycin F4a in gastric cancer cells [53], a specific ATG4B inhibitor named DC‐ATG4in in hepatocellular carcinoma cells [54], and tioconazole in CRC cells [55]. Although the roles of ATG4C and ATG4D in autophagy are less well‐characterized compared to ATG4A and ATG4B, knockdown of *ATG4C* has been shown to induce cell cycle arrest at the G1 phase and ROS‐mediated apoptosis in glioma cells, likely through suppression of autophagy [50]. Depletion of ATG4C also attenuated temozolomide‐induced autophagy and increased the chemosensitivity of glioma cells. However, the role of ATG4D in cancer cell proliferation and drug resistance, particularly in osteosarcoma cells, remains poorly understood. This study suggested that silencing ATG4 family members (*ATG4A, ATG4B, ATG4C*, and *ATG4D*) led to an increased proportion of cells in the G1 phase in osteosarcoma cells. Knockdown of *ATG4D* significantly elevated the subG1 population, accompanied by an increase in caspase‐3/7 activity. ATG4D ablation also sensitized osteosarcoma cells to starvation and chemotherapeutic treatments. In addition, we analyzed the mRNA expression levels of *ATG4* family members in osteosarcoma cell lines 143B and U2OS (Figure S2). Among the four isoforms, *ATG4B* and *ATG4C* exhibited relatively higher transcript levels, while *ATG4D* expression was comparatively lower. Notably, these mRNA expression patterns did not fully correlate with the phenotypic effects observed upon gene silencing, implying that ATG4D's functional role in osteosarcoma may depend more on its protein abundance or posttranslational regulation. Further investigation is warranted to clarify the mechanistic contribution of ATG4D to osteosarcoma oncogenesis. Nevertheless, these results suggest that ATG4D is essential for cell cycle progression in the G1 phase and plays a cytoprotective role under stress conditions, such as starvation and chemotherapeutic drug exposure.

Several studies have reported a correlation between increased levels of the autophagy marker LC3B and metastasis in various cancer types, including breast cancer, melanoma, hepatocellular carcinoma, and glioblastoma [43]. These findings suggest that autophagy plays a role in promoting cancer metastasis and enhancing the aggressiveness of cancer cells. Overexpression of ATG4A has been shown to promote the epithelial‐mesenchymal transition (EMT) phenotype and stem‐like properties in gastric cancer cells, characterized by reduced E‐cadherin levels and elevated Sox‐2 expression. Conversely, silencing *ATG4A* reverses these effects on signaling molecules [56]. Additionally, ATG4A overexpression significantly enhanced gastric cancer cell migration and invasion in vitro and promoted metastasis in vivo, likely through the activation of Notch signaling pathways. Furthermore, silencing *ATG4B* has been shown to inhibit cell migration and invasion in CRC cells [12]. Consistent with previous reports, knockdown of *ATG4* family members significantly reduced the motility of osteosarcoma cells, particularly in *ATG4D*‐silenced cells. These results suggest that ATG4D may play a pivotal role in cancer metastasis, highlighting the need for further investigation to elucidate its exact mechanisms.

ATG4 plays a pivotal role in the proteolysis of ATG8 orthologues, cleaving the C‐terminal glycine residue to facilitate their conjugation with phosphatidylethanolamine (PE) during autophagosome formation [57]. In vitro assays have demonstrated that ATG4B exhibits the highest proteolytic activity among the four ATG4 family members, while ATG4A preferentially targets the GBRP superfamily [24, 25]. However, single knockout *ATG4B* or double knockout of *ATG4A/ATG4B* cannot completely block LC3/GABARAP processing and autophagy in HeLa cells [45], suggesting each ATG4 member can compensate for the autophagic function of the other ATG4 members in cells. Both ATG4C and ATG4D contain a caspase‐3 cleavage site at their N‐terminus, and these proteins are cleaved by caspase‐3 to activate their proteolytic activity [23]. Cleaved ATG4D is particularly associated with apoptosis due to its BH3 domain, which exposes it to disrupt the antiapoptotic protein Bcl‐2 during mitochondrial damage [23]. Interestingly, ATG4C and ATG4D are also involved in the priming and lipidation of GABARAP isoforms, independent of caspase‐3 expression in HeLa cells [45]. Recent studies have further highlighted that ATG4s, along with their partner protein LPS‐responsive beach‐like anchor protein (LRBA), regulate ATG9A vesicle trafficking, promoting the formation of phagophore‐ER contacts for phagophore expansion during mitophagy in HeLa cells, in a protease‐independent manner [44]. Furthermore, while ATG4s are not essential for the removal of ATG8 from autolysosomes, they are required for the deconjugation of ATG8ylation (ATG8 on proteins) during mitophagy induction in HeLa cells [44]. In contrast, Atg4d knockout results in defects in the removal of lipidated LC3B from the cytosolic leaflet of autophagosomal and autolysosomal membranes in murine cells, a defect not observed in *Atg4a*, *Atg4b*, or *Atg4c* null mutants [29, 58]. These findings are consistent with observations in HCT116 *ATG4D* ^−/−^ cells and *Atg4d* ^−/−^ mice, which accumulate lipidated LC3, GABARAPL1, and GABARAPL2 [29, 59]. This study revealed that *ATG4D* knockdown increased LC3B‐II puncta (lipidated LC3) and inhibited autophagy, sensitizing osteosarcoma cells to starvation conditions. However, further investigation is required to evaluate the specific role of ATG4D protease activity and ATG8ylation in autophagy and drug resistance in osteosarcoma cells. In addition, our study showed a correlation between ATG4D protein levels and poor survival outcomes in patients with osteosarcoma. However, the small sample size (*n* = 86) in our cohort may limit the ability to fully account for potential confounders, particularly in the analysis of stratified subgroups. To confirm these findings and enhance the robustness of the evidence, a larger, more diverse cohort would be required. Despite these limitations, our results align with observations in cell culture, suggesting that the associations identified may be valid.

Although recent studies indicate that several potential prognostic and therapeutic biomarkers for patients with osteosarcoma [60, 61], the role of ATG4 family members in osteosarcoma is not known. Moreover, previous reports have identified potential inhibitors for ATG4B and ATG4A as mentioned above. Nevertheless, no specific inhibitors for ATG4D have been reported, likely due to the limited assays available for ATG4D. Our study suggests that ATG4D plays a crucial role in the tumor malignancy of osteosarcoma. The siRNA approach employed in this study may offer a promising therapeutic strategy for osteosarcoma in future developments.

## Author Contributions

C.‐W.S. and P.‐F.L. conceived the study, confirmed the authenticity of all the raw data, and reviewed the manuscript. S.‐W.Y., C.‐H.L. and W.‐H.Y. analyzed data. S.‐W.Y., W.‐H.Y., S.‐F.H., C.‐Y.T., C.‐J.H., and H.‐H.T. performed experiments and interpreted data. P.‐F.L., S.‐W.Y., and C.‐W.S. designed the experiments. C.‐W.S. and P.‐F.L. wrote the manuscript. All authors have read and approved the final manuscript.

## Ethics Statement

The animal study was reviewed and approved by the Ethics Committee of the National Sun Yat‐sen University.

## Consent

The authors have nothing to report.

## Conflicts of Interest

The authors declare no conflicts of interest.

## Supporting information


**Figures S1–S2:** fsb270990‐sup‐0001‐FiguresS1‐S2.pdf.

## Data Availability

The data generated in this study are included in the figures and/or tables of this article.
