# Supplementary material for: Autophagy Protease ATG4D Facilitates Proliferation and Malignancy of Osteosarcoma Cells
Source: FASEB J. 2025 Aug 29;39(17):e70990. doi: 10.1096/fj.202501321RR (PMC12397559; doi:10.1096/fj.202501321RR)
Supplement: Supplementary file 1 — Figures S1–S2: fsb270990‐sup‐0001‐FiguresS1‐S2.pdf. [file FSB2-39-e70990-s001.pdf]

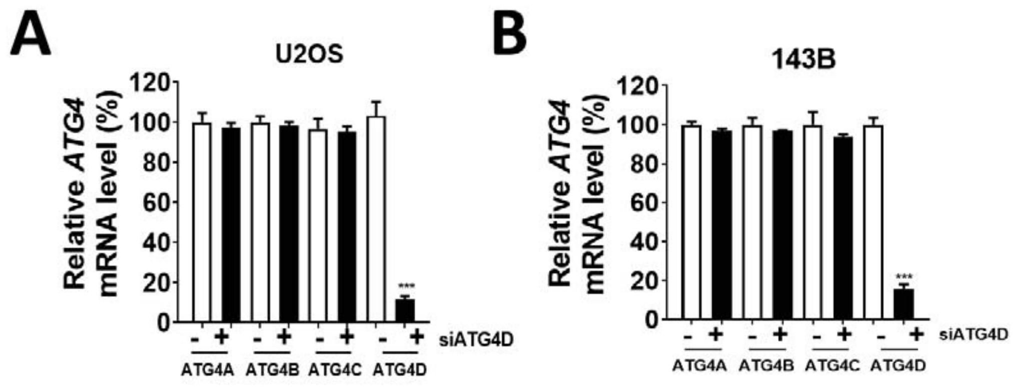

Figure S1. The silencing specificity of si*ATG4D* on *ATG4* isoforms gene expression in osteosarcoma cells

(A) U2OS and (B) 143B osteosarcoma cells were transfected with 5 nM siRNA targeting *ATG4D* for 48 hrs. The mRNA expression levels of each *ATG4* isoform were quantified using qPCR to confirm knockdown specificity of si*ATG4D*.

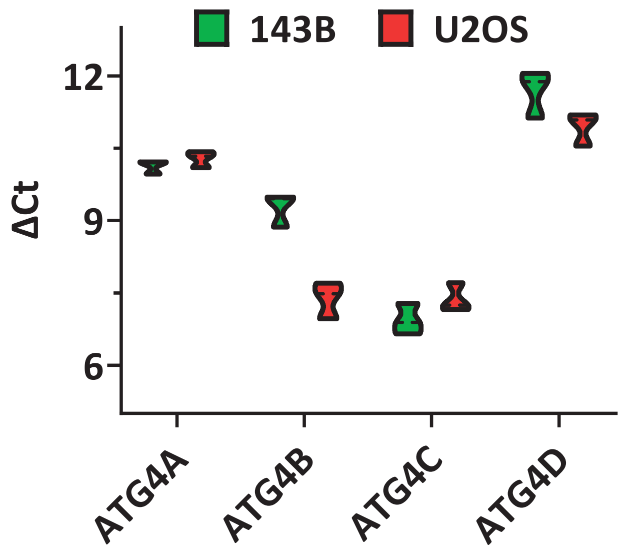

Figure S2. The effects of *ATG4* gene expression levels in osteosarcoma cells.

U2OS and 143B osteosarcoma cells were cultured and harvested to isolate mRNA. The mRNA expression levels of each *ATG4* isoform were quantified using qPCR.
